# Influence of splenectomy on immunoglobulins and complement components in major thalassemia

**Published:** 2015

**Authors:** Ali Asghar Darzi, Sakineh Kamali, Mohammad Khakzad

**Affiliations:** 1 Department of Surgery, Babol University of Medical Sciences, Babol, Iran.; 2 Shahid Beheshti Hospital, Clinical Research Development Center, Babol University of Medical Sciences, Babol, Iran.

**Keywords:** Major thalassemia, Splenectomy, Immunoglobulin, Complement

## Abstract

**Background:**

Thalassemia is the most common anemia with hereditary base in Iran. The aim of this study was to evaluate the humoral immune system and assess the effect of splenectomy on the serum level of immunoglobulins IgG, IgM, and IgG and complement components in patients with major thalassemia.

**Methods:**

This quasi-experimental study (before-after) was performed on 40 patients with major thalassemia that referred to the treatment centers of Babol for splenectomy from March 2011 to March 2013.

**Results:**

The mean age of patients under study was 25.38±6.89 years. The results of this study showed that the rate of IgA and IgM had a significant decrease after splenectomy. However, the increase of serum level in IgG in this study was not significant. The serum levels of C_3_ and CH_50_ were evaluated in all patients that its reduction was statistically significant, but the decrease of serum levels in C_4_ was not significant.

**Conclusion:**

The spleen plays a role in releasing immunoglobulins and starter proteins of complement activation pathways and splenectomy causes reduction in the serum levels of immunoglobulins and complement components.

Thalassemia is one of the most prevalent hematologic disorders worldwide (1, 2). It is the most common inherited anemia in Iran and in the world is thalassemia and the most common genetic disease in Mazandaran province is beta-thalassemia (3, 4). Paleness, the lack of weight gain, impairment in growth and abdominal enlargement are the most common symptoms of the disease (5). Destruction of red blood cells and ineffective erytheropoiesis are the major causes of anemia. A combination of both ineffective hematopoiesis and severe anemia in patients with major thalassemia causes skeletal deformity, enlargement of liver and spleen (1, 5). The management of major thalassemia includes the administration of drugs for blood formation, packed cell transfusion and prevention of iron storage disease (6). 

Splenectomy is advised when transfusion volume exceeds 249 ml/kg of body weight per year (7). The risk of sepsis in splenectomized patients is as high as 7% over a 10-year period and almost 25% of splenectomised patients are at risk of severe infections (8) which is attributed to the quantitative and qualitative abnormalities in the production of immunoglobulins, impaired activity of T and B cell lymphocytes, dysfunction of macrophages and neutrophils, as well as impairment in the components of the complement system (9, 10).

Several studies showed increased levels of serum immunoglobulin G (IgG) and immunoglubolin A (IgA), but in our study it showed stable or increased levels of serum immunoglobulin M (IgM) in splenectomized major thallassemia (11-15). Several studies reported that the level of IgG and IgA in serum in patients increased normally and the level of IgM was normal or decreased (11-15). However, the data regarding serum immunoglobulin status in major thallassemia patients undergoing splenectomy are scarce. This issue is of particular importance specifically in the geographical regions of Mazandaran where they provide health services for thalassemic patients and a substantial number of them are managed by regular transfusion and may require splenectomy in the future. 

For these reasons, the present study was designed to determine the influence of splenectomy on serum immunoglobulins and complement the patients with major thalassemia who underwent splenectomy.

## Methods

The study population consisted of 40 thalassemia major patients who underwent splenectomy. These patients were referred from Amirkola Thalassemia Research Center to Shahid Beheshti Hospital, Babol, Iran between March 2011 to March 2013.

Informed consent was provided by all participants. The data regarding patient characteristics and disease process were provided through a review of the patients’ medical records. The patients’ cell blood count and serum IgG, IgA, IgM, C3, C4, and CH50 were determined at baseline before splenectomy and three months after surgery. The objective of this study was to determine the mean changes from baseline in serum IgG, IgA, IgM, C3, C4 three months after splenectomy by the comparrison of serum levels of these variables using paired t-test.

## Results

Twenty- six men and 14 women with mean age of 25.92±5.97 and 24.36±8.49 years were analyzed. Three months after splenectomy, the serum levels of C3, IgA, IgM decreased significantly as compared with preparation levels (P=0.007, P=0.001, and 0.001, respectively) (table 1). There was also a significant decrease in CH50 (P=0.01) and a nonsignificant decrease in C4 from baseline (P=0.0.054) but there was no change in serum IgG level as compared with baseline level (P=0.48). The influence of splenectomy varied according to sex. In female patients, serum complement components did not change from baseline, however, in males, splenectomy resulted a significant decrease in serum C3 and CH50 (P=0.033, and P=0.017, respectively). However, variations in serum immunoglubolins did not differ between the two sexes (table 2). 

**Table 1:** The mean levels of serum immunoglobulins and complement components before and after 3 months surgery

**P**	**After** **Mean±SD**	**Before** **Mean±SD**	
P=0.587	1584.15±324.44	1571.96±369.25	IgG
P<0.001	170.48±71.96	192.04±87.71	IgM
P<0.001	256.05±97.99	269.99±103.34	IgA
P=0.007	101.08±22.37	106.80±22.79	C3
P=0.054	18.55±4.88	19.47±6.21	C4
P=0.010	81.70±22.56	86.92±27.66	CH50

**Table 2:** The mean levels of serum immunoglobulins and complement components according to sex

**Men**		**Women**		
**baseline**	**P**	**Endpoint**	**P**	
328.39±19.16	P=0.838	520.28±29.16	P=0.493	IgG
983.90±04.20	P<0.001	496.63±71.16	P=0.017	IgM
609.11±97.28	P=0.001	071.82±36.24	P=0.020	IgA
876.27±58.11	P=0.033	521.27±0.10	P=0.82	C3
878.5±82.18	P=0.207	458.5±36.19	P=0.154	C4
972.28±02.86	P=0.017	641.24±64.85	P=0.333	CH50

## Discussion

The results of this study indicate that splenectomy has a significant decline in serum IgA, IgM but no change in IgM levels in both male and female patients with major thalassemia. Furthermore, in males with thalassemia, but not in females with splenectomy decrease C3, and CH50 significantly as compared with baseline level. However, no changes were observed in C4 levels in both sexes. 

The findings of this study are consistent with other studies with regard to IgM, and IgM status after splenectomy (16, 17). However, the results of this study are in contrast with other studies. Ahluwalia et al. did not detect any influence of splenectomy on serum immunoglobulins (18). In another study from Iran, Karimi et al. found higher IgG in splenctomized thalassemia than controls. In addition, they found no significant differences in IgA. IgM, C3, C4 between patients with and without splenectomy (13).

The differences across various studies should be attributed to several factors including, age, sex, duration and the magnitude of transfusion and the genetic background of the study population. The spleen plays an important role in the release of immunoglobulins, mainly IgM which is the major source of protein properdin. Splenectomy reduces serum immunoglobulins and complement components and increases the amount of infection, including sepsis (19). The results of our study support the existing data indicating a predisposition of splenectomized thalassemic patients to infection. However, splenectomy exerts beneficial effects for these patients including reduction in blood transfusion, an improvement of anemia.

In conclusion, this study indicates that splenectomy in patients with major thalassemia results in the significant reduction of immunoglobulins and complement and imposes these patients at greater risk of infection.

## Notes

**Funding:** This study was funded by Deputy of Research and Technology of Babol University of Medical Sciences.
